# The Association between Dry Eye Disease and Physical Activity as well as Sedentary Behavior: Results from the Osaka Study

**DOI:** 10.1155/2014/943786

**Published:** 2014-11-17

**Authors:** Motoko Kawashima, Miki Uchino, Norihiko Yokoi, Yuichi Uchino, Murat Dogru, Aoi Komuro, Yukiko Sonomura, Hiroaki Kato, Yuji Nishiwaki, Shigeru Kinoshita, Kazuo Tsubota

**Affiliations:** ^1^Department of Ophthalmology, Keio University School of Medicine, 35 Shinanomachi, Shinjuku-ku, Tokyo 1608582, Japan; ^2^Department of Ophthalmology, Kyoto Prefectural University of Medicine, Kyoto, Japan; ^3^Department of Environmental and Occupational Health, Faculty of Medicine, Toho University, Japan

## Abstract

*Purpose*. To assess the association of dry eye disease (DED) with physical activity and sedentary behavior.* Methods*. The cross-sectional survey conducted included Japanese office workers who use visual display terminals (*n* = 672). DED was assessed according to the Japanese Dry Eye Diagnostic Criteria, and participants were categorized into “definite DED,” “probable DED,” or “non-DED” groups based on the results of DED examinations. Physical activity and sedentary behavior of participants were assessed using the International Physical Activity Questionnaire (IPAQ), and physical activity level was calculated in metabolic equivalent units per week (MET, min/week). Participants were classified as having a high, moderate, or low level of physical activity.* Results*. Participants with abnormal tear break-up time (BUT) (≤5 s) were involved in sedentary behavior for significantly longer duration than those with normal BUT (*P* = 0.035). Non-DED participants (14.5%) tended to have higher levels of physical activity than definite DED participants (2.5%). Participants with definite DED had significantly lower MET scores than those with non-DED (*P* = 0.025).* Conclusions*. Our findings suggest that a lower level of physical activity and sedentary behavior are associated with DED; however, longitudinal/intervention studies with large groups of participants are needed to validate these findings.

## 1. Introduction

Dry eye disease (DED) is well known as a multifactorial chronic disorder that is highly prevalent in many countries, including Japan [1, 2]. DED presents with various symptoms, and the disease can significantly affect the quality of life (QOL) of patients. DED has physical, social, and psychological consequences because it results in impaired visual function and/or psychological problems [3–8]. In addition, DED has become an important public health problem because of its high prevalence in the general population.

The use of visual display terminals (VDT), including laptops, electronic tablets, and readers, as well as smartphones, is considered an important cause of DED. It is well known that work with VDT can have a wide range of psychological and physiological adverse effects, including symptoms of DED. Office workers tend to use VDT often [9], and hence, DED is more prevalent amongst them. It has been reported that the development of DED can lead to deterioration in work performance [10]; therefore, DED should be an important consideration in the management of office workers. In addition, maintaining the mental health of office workers has been recognized as a critical component in office management. Recent studies reported that, in addition to low levels of physical activity, a prolonged period of sedentary behavior (sitting for instance) is a risk factor for various health problems, including chronic diseases such as cardiovascular disease, diabetes, and metabolic syndrome [11–13]. Because physical inactivity and a sedentary life style are detrimental to health, addressing these behaviors has become a global public health priority [14]. Office workers who tend to sit for long periods of time generally suffer from lower back pain and/or painful eyes [15, 16]. Eye strain is an important contributor to the development of DED—a disease that has been neglected as a component in the health management of office workers. Our previous cross-sectional studies have confirmed that VDT work is a risk factor for DED [17]. We also speculate that physical activity and sedentary behavior are associated with DED, and the present study aimed at assessing the impact of sedentary behavior and physical activity on DED among VDT workers.

## 2. Methods

We analyzed the data from a cross-sectional study that was conducted in 2011 amongst 672 office workers aged between 26 and 64 years, who were employed at a company in Osaka, Japan (the Osaka study) [17]. We investigated the association between DED parameters (Schirmer's test *I* values, tear film break-up time (BUT) test values, ocular surface staining scores, and symptoms) and physical activity level as well as sedentary behavior. Detailed methods used in this study have been described previously [17]. Briefly, written informed consent was obtained from all participants. Candidates with a history of refractive surgery were excluded from the study. The research protocol was in accordance with the Declaration of Helsinki, and it was based on a protocol approved by the Institutional Review Board of the Ryogoku Eye Clinic Tokyo, Japan.

### 2.1. Diagnosis of Dry Eye Disease

Participants were classified into the following categories based on the results of dry eye examinations, including Schirmer's test, fluorescein and lissamine green staining, tear film BUT, and a completed symptom questionnaire: “definite DED,” “probable DED,” and “non-DED.” A diagnosis of DED was made according to the latest Japanese Dry Eye Diagnostic Criteria (2006), which lists the following symptoms: (1) the presence of dry eye symptomatology, (2) the presence of qualitative or quantitative disturbance of the tear film (Schirmer's test ≤ 5 mm or BUT ≤ 5 s), and (3) the presence of keratoconjunctival epithelial damage (total score of fluorescein and lissamine green staining ≥3 points). If all 3 criteria were met, a diagnosis of “definite DED” was made. Participants who fulfilled 2 of the 3 criteria were categorized as “probable DED,” and those who met only 1 or none of the 3 criteria were categorized into the “non-DED” group [18, 19].

### 2.2. Physical Activity Level

The short form of the International Physical Activity Questionnaire (IPAQ-J) was used to determine physical activity during leisure time, domestic work, paid or unpaid work, and transport [20, 21]. The participants were questioned on the following 3 specific types of physical activity, in which they participated at any time during their daily routine: walking, moderate-intensity activity, and vigorous-intensity activity. Scores for each type of activity were calculated by summing the scores for duration and frequency. The volume of activity was then calculated by weighting each type of activity by its energy requirements. This was defined in metabolic equivalent units (MET), and we calculated a MET score per week (MET, min/week). Total MET (in min/week) was calculated by summing all the scores for each type of activity. Finally, the level of physical activity (high, moderate, or low) was based on the calculated scores.

The IPAQ question regarding sitting behavior can be considered an additional indicator of the amount of time spent in sedentary activity, and it was not included in the calculations of physical activity. In this study, the number of hours per day spent sedentarily was obtained from the self-reported total time spent sitting as assessed by the IPAQ (e.g., sitting at a desk, watching television, and reading).

### 2.3. Statistical Analysis

Categorical variables were analyzed using chi-square tests. Continuous variables were analyzed using analyses of variances (ANOVA) and Tukey's multiple comparisons. The association between continuous variables was investigated with Pearson's correlation. Multiple regression analysis was used to estimate the impact of IPAQ on DED after adjustments for the possible confounders (gender, age, and VDT hours). To eliminate age as a confounding variable, we stratified all major analyses by age over 30 years and adjusted for age, gender, and VDT hours. Values of *P* < 0.05 were considered significant. All statistical analyses were performed using SAS software, version 9.2 (SAS Inc., Cary, NC).

## 3. Results

### 3.1. Physical Activity and DED

Characteristics of the participants are summarized in Table 1. The survey response rate for the completion of the IPAQ, dry eye questionnaires, and dry eye examinations was 63.2% (425 of 672 participants). The average amount of time spent using VDT tended to be long when the DED criteria were considered; however, there was no significant difference between the DED status and VDT usage (*P* = 0.093).

**Figure 1:**
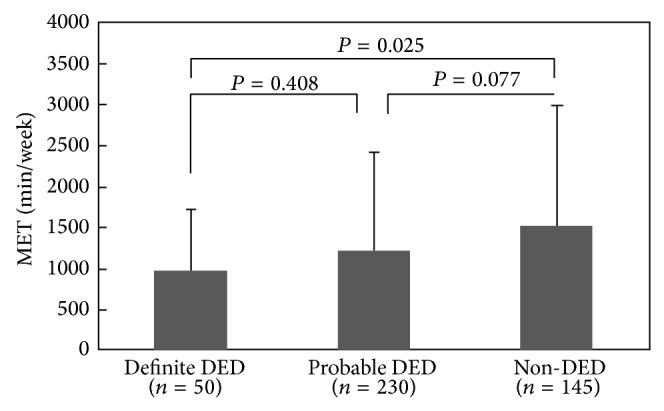
Comparative analysis of MET score and dry eye disease. Each column shows the mean ± SD. *P* values were analyzed by Tukey's multicomparisons following ANOVA. DED: dry eye disease.

The physical activity levels and MET scores according to DED criteria are shown in Table 2 and Figure 1, respectively. According to the IPAQ data, 10.1%, 48.7%, and 41.2% of participants engaged in high, moderate, and low levels of physical activity. Participants in the non-DED group (14.5%) tended to score higher in physical activity than those in the definite DED group (2.5%). The mean MET scores of the non-DED and the definite DED groups differed significantly (*P* = 0.025) (Figure 1). The correlation between the level of physical activity and clinical findings of DED are shown in Table 3. The IPAQ score was significantly correlated with the tear film BUT scores and the ocular surface epithelial staining scores, but not with Schirmer's test *I* values.

Furthermore, we assessed the association between MET score and the diagnostic criteria of DED after accounting for factors known to be associated with DED (age, gender, and VDT hours), and we found a significant association between the diagnostic criteria of DED and MET scores (*P* = 0.015).

We also investigated whether sedentary behavior was associated with DED. Although we could not find a significant association between a diagnosis of DED and sedentary behavior, our results show that the participants with abnormal BUT (≤5 s) were involved in sedentary behavior for a significantly longer duration than those with normal BUT (>5 s) (*P* = 0.035, Table 4).

## 4. Discussion

Ocular symptoms such as eye dryness, eye strain, and blurry vision are symptoms commonly reported by VDT users [22]. A large-scale epidemiologic study amongst office workers in Japan recently showed that VDT work is an important risk factor for DED [9]. With the rapid advances in information technology, VDT use amongst office workers has increased considerably in recent years. We conducted this study to understand the actual health status of office workers, specifically focusing on DED, which is a multifactorial disease that is not yet well known.

The present cross-sectional study confirmed that DED is highly prevalent, occurring in about 12% of office workers who spend, on average, 8 h per day working with VDT. VDT-related DED is thought to result from lower blinking rates and an increased tear evaporation rate with tear film instability [23]. However, with DED being a multifactorial disease, these are not the exclusive causes of this disorder. We hypothesized that a sedentary lifestyle contributes to DED. DED as well as many other painful symptoms including shoulder and/or neck pain can be related to the sedentary lifestyle syndrome, which is a major work-related health problem amongst workers with sedentary jobs [24].

Recently, a decrease in physical activity has been earmarked as a serious public health concern in developed countries, because it is thought to induce a number of diseases including cardiovascular diseases and metabolic syndrome. Recent data also suggests that sedentary behaviors, along with the decrease in physical activity, might be important in the etiology of type II diabetes mellitus and metabolic syndrome [11–13]. The association between DED and sedentary behavior, which is defined as engaging in activities at the resting level of energy expenditure and includes sleeping, sitting, lying down, using a computer, and viewing television, has not been studied thus far.

Our results show that a higher level of physical activity is associated with a lower risk of DED and that sedentary behavior is associated with DED. Tear film BUT and ocular surface staining scores were significantly associated with IPAQ scores (Table 3); further, BUT was significantly associated with sedentary time (Table 4). To our knowledge, this is the first study to report these associations.

A life-style with physical activity might influence the qualitative and/or quantitative characteristics of tear film. Recent studies have investigated the relation between dry eye symptoms and a short BUT (which can be recognized by an unstable tear film) [25, 26], and this type of DED seems to involve a large number of patients with DED. In total, 78.6% of the participants in the cross-sectional study had abnormal tear film BUT values (≤5 s), and the majority of those with probable DED experienced DED symptoms and abnormal BUT [17]. One of the factors related to tear film instability is reduced mucin expression [27]. Physical inactivity and sedentary behavior induce various chronic disorders including systemic inflammation-related diseases [28], which are thought to be related to excess oxidative stress. Therefore, it may also induce ocular surface inflammation, increase oxidative stress, reduce mucin expression, and result in short BUT type dry eye.

From these results, we conclude that an increase in the level of physical activity can be an effective intervention for the prevention and/or treatment of DED. Further, there is considerable evidence suggesting that exercise could improve both mental and physical health [29, 30]. Thus far, several cross-sectional studies have shown that a higher level of physical activity results in an improved mental health status [31]. It has been reported that physical exercise interventions attenuate the intensity of headache as well as neck and shoulder symptoms and upper extremity muscular strength in office workers [16].

Therefore, exercise can be a beneficial intervention strategy for DED, in addition to adequate medical treatment such as eye drops. These interventions can improve eye condition and, at the same time, alleviate other diseases, including pain in the neck, shoulders, and/or low back, as well as depression. An intervention that would increase the physical activity level of office workers may well contribute to improvements in the QOL of office workers, especially when considering that DED is known to negatively affect QOL [3–8] and productivity [10]. It is clearly important to enhance awareness of DED not only amongst office workers but also amongst industrial doctors.

Moss et al. reported that a sedentary life style has a lower risk of dry eye incidence, which contradicts our findings [32]. However, a sedentary lifestyle in that study was identified with an interview, and the characteristics of participants, including age range and the frequency of VDT work, differed from those of the participants in our study.

Our study had some limitations. First, the conduct of the current study is flawed by a relatively low sample size to draw any definitive conclusions. Second, this study was done by the inclusion of Japanese patients only. It may be difficult to generalize to the other population. Thirdly, there was a potential selection bias; our study had a low response rate (63.2%), which could have introduced a bias for a selected group of participants. Forth, we used a self-reported questionnaire to assess physical activity and sedentary behavior, which could have been completed subjectively by participants. We suggest that future studies use objective tools such as an accelerometer to evaluate physical activity. Lastly, as this was a cross-sectional study, causality for DED remains unresolved. In order to validate our findings, we need larger longitudinal/interventional trials in near future.

In conclusion, the results of the present study showed that physical activity and sedentary behavior are associated with DED, and higher levels of physical activity may lower the risk of DED. Further longitudinal or intervention studies in large study groups are necessary to improve our understanding of DED.

## Figures and Tables

**Table 1:** Characteristics of the study participants who completed dry eye disease examinations and a physical activity questionnaire.

		Definite DED	Probable DED	Non-DED	Total	*P* value
*N*		50	230	145	425	—
Sex *N* (%)	Men	23 (46.0)	143 (62.2)	114 (78.6)	280 (65.9)	0.000^(1)^
	Women	27 (54.0)	87 (37.8)	31 (21.4)	145 (34.1)	

Age	Mean ± SD	41.0 ± 7.2	42.5 ± 8.3	43.1 ± 8.9	42.5 ± 8.4	0.323^(2)^

Age (category) *N* (%)	20 s	3 (6.0)	12 (5.2)	13 (9.0)	28 (6.6)	0.192^(1)^
	30 s	18 (36.0)	73 (31.7)	32 (22.1)	123 (28.9)	
	40 s	22 (44.0)	98 (42.6)	62 (42.8)	182 (42.8)	
	50 s	7 (14.0)	47 (20.4)	38 (26.2)	92 (21.6)	

VDT time	Mean ± SD	8.4 ± 2.2	8.1 ± 2.3	7.7 ± 2.1	8.0 ± 2.2	0.093^(2)^

VDT time (2 categories) *N* (%)	<8 h ≥8 h	34 (68.0) 26 (17.9)	163 (70.9) 67 (29.1)	119 (82.1) 16 (32.0)	316 (74.4) 109 (25.6)	0.029^(1)^

^(1)^ *χ* ^2^ test ^(2)^ANOVA, DED: dry eye disease, VDT: visual display terminal, and SD: standard deviation.

**Table 2:** Comparison of physical activity according to dry eye disease criteria.

		Definite DED	Probable DED	Non-DED	Total
*N*		50	230	145	425
Physical activity assessed by IPAQ	High (*n* (%))	2 (2.5)	20 (8.7)	21 (14.5)	43 (10.1)
	Moderate (*n* (%))	27 (54.0)	111 (48.3)	69 (47.6)	207 (48.7)
	Low (*n* (%))	21 (42.0)	99 (43.0)	55 (37.9)	175 (41.2)

IPAQ: International Physical Activity Questionnaire, DED: dry eye disease, and SD: standard deviation.

**Table 3:** Pearson's correlation between International Physical Activity Questionnaire score and the clinical findings of dry eye disease.

Dry eye clinical findings	Mean ± SD	Pearson's correlation coefficient	*P* value
Ocular surface staining score	1.1 ± 1.3	−0.153	0.002
Schirmer's *I* test value (mm)	18.9 ± 11.7	0.011	0.822
BUT (s)	4.1 ± 2.6	0.117	0.012

BUT: tear film break-up time and SD: standard deviation.

**Table 4:** Sedentary behavior, DED diagnosis, and clinical parameters.

	*N*	Sedentary time (min) (mean ± SD)	*P* value^(1)^
Dry eye diagnosis			
Definite DED	50	575.4 ± 244.1	0.551
Probable DED	227	582.9 ± 257.4	
Non-DED	140	552.9 ± 260.8	
Schirmer's test *I* value			
<5 mm (abnormal)	67	531.9 ± 276.9	0.164
≥5 mm	350	579.6 ± 252.4	
BUT			
≤5 s (abnormal)	325	586.0 ± 253.7	0.035
>5 s	92	522.1 ± 262.8	
Ocular surface staining score			
<3	346	574.0 ± 262.4	0.709
≥3 (abnormal)	71	561.5 ± 229.0	

Total	417	571.9 ± 256.8	

^(1)^ANOVA, DED: dry eye disease, BUT: tear film break-up time, and SD: standard deviation.
